# Sex differences in vancomycin-resistant enterococci bloodstream infections—a systematic review and meta-analysis

**DOI:** 10.1186/s13293-021-00380-5

**Published:** 2021-05-17

**Authors:** Carlos L. Correa-Martínez, Franziska Schuler, Stefanie Kampmeier

**Affiliations:** 1 Institute of Hygiene, University Hospital Münster, Robert-Koch-Strasse 41, 48149 Münster, Germany; 2 Institute of Medical Microbiology, University Hospital Münster, Domagkstrasse 10, 48149 Münster, Germany

**Keywords:** Vancomycin-resistant enterococci, VRE, Bloodstream infection, Bacteremia, epidemiology, Sex differences

## Abstract

**Background:**

Vancomycin-resistant enterococci (VRE) have emerged in the healthcare setting worldwide. Infections with these pathogens, i.e., bloodstream infections (BSI), are accompanied with an impaired patient outcome. Diverse factors comprising patient characteristics, therapeutic strategies, and infection control measures are positively or negatively associated with VRE BSI occurrence. However, whether sex-specific differences influence the frequency of VRE BSI is yet unknown.

The aim of this systematic review was to comprehensively summarize and analyze sex prevalence in VRE BSI patients.

**Main text:**

A systematic search for relevant articles was conducted in PubMed and Web of Science. After screening for eligibility, data extraction from included articles and risk of bias assessment were processed. The prevalence of male/female sex in VRE BSI patients and 95% CI were calculated for each study and summarized as pooled estimated effect.

In total, nine articles met the inclusion criteria. Risk of bias assessment resulted in low (six studies) to moderate bias (three studies). The pooled prevalence of male patients suffering from VRE BSI was 59% resulting in a 1.4 male/female prevalence ratio.

**Conclusions:**

Current literature suggests sex differences with male preference (59%) in the distribution of VRE BSI cases. Further primary studies should address the question of male-specific factors favoring the enhanced frequency of VRE BSI.

## Highlights

- Sex differences play a role in the emergence of infectious diseases.
- The overall prevalence of male patients suffering from VRE BSI is 59%.
- Male/female ratio in VRE BSI is 1.4.

## Background

Since their first description in the 1980s [1, 2], vancomycin-resistant enterococci (VRE) have evolved to become some of the most relevant multidrug-resistant organisms (MDRO) worldwide as acknowledged by the World Health Organization [3]. VRE pose a particular challenge for healthcare settings, given their ability to survive in the environment [4, 5] as well as their higher nosocomial prevalence as compared to other MDRO [6]. Besides colonizing the gastrointestinal tract of their human host, VRE may cause, inter alia, abdominal, foreign body-associated, and bloodstream infections (BSI) [7]. Invasive VRE infections, especially BSI, are known to have a higher mortality than those caused by vancomycin-susceptible enterococci [8]. Among VRE associated with human disease, *Enterococcus faecium* (*E. faecium*) represents the most relevant species, accounting for over 93% of all VRE isolates in Europe in 2019 [9]. The detection of VRE in clinical samples has continuously increased in several regions [10]. In Europe, this information has been systematically collected and monitored by the European Centre for Disease Control and Prevention (ECDC) since 2015, analyzing the proportion of vancomycin resistance among *E. faecium* strains isolated from blood cultures as a benchmark for comparison between countries [11]. Starting at an average of 10.5% in ECDC’s first regional data analysis in 2015, this proportion has steadily increased over the last years, reaching 17.3% in 2018, with rising trends in over 20 of the 30 countries evaluated [11].

**Fig. 1:**
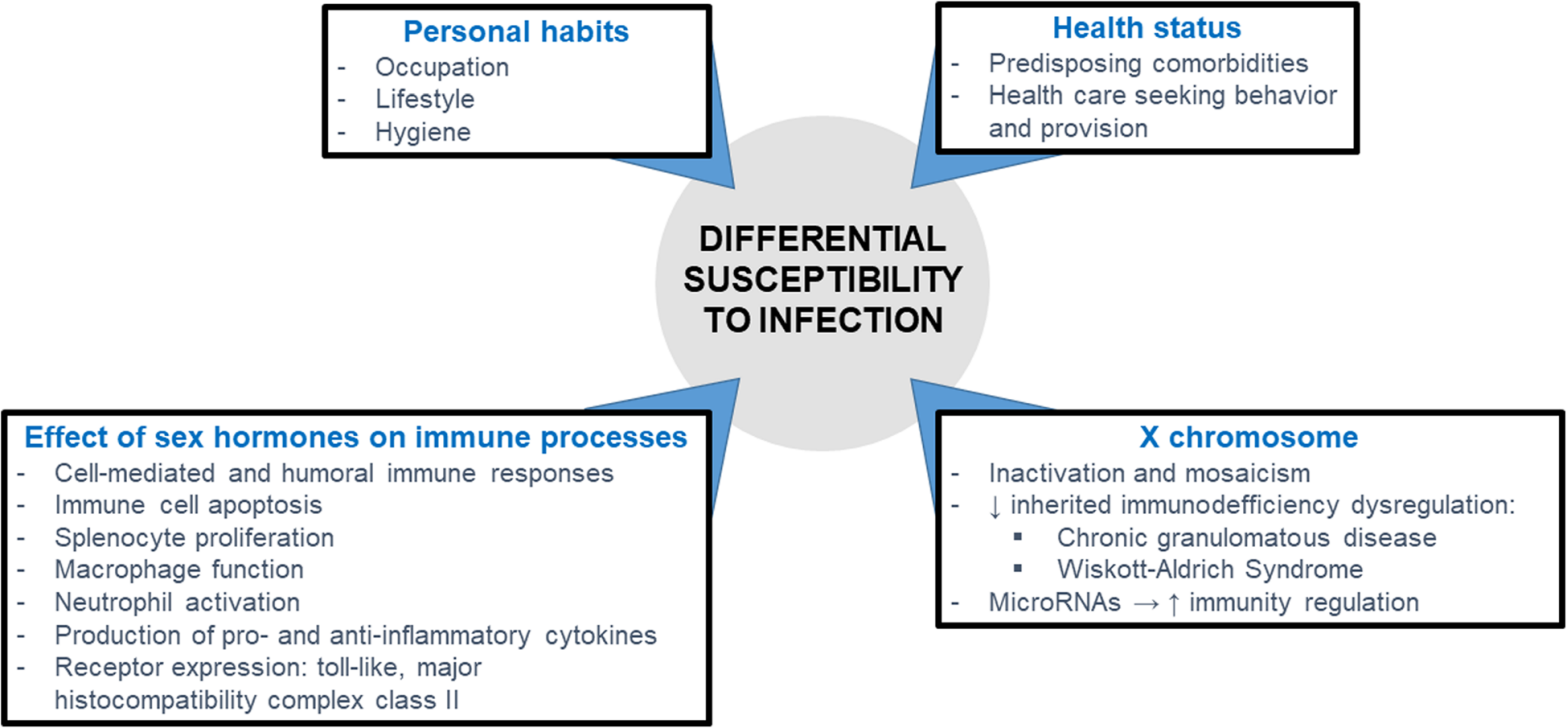
Sex differences influencing the occurrence of infectious diseases

Several factors have been described to be significantly associated with VRE colonization and infection, including treatment with antibiotics, immunosuppression, and further pre-existing pathologies and treatments [12–14]. Regarding demographic characteristics, the incidence of VRE infections is higher in patients of older age [15, 16]. However, it is unclear whether an association exists between sex and the development of VRE infections. Sex differences in the occurrence of numerous infectious diseases have been conclusively described (Fig. 1), having been linked with hormonal and genetic factors that contribute to this phenomenon [17, 18]. Among others, these sex differences have been described to play a role in the composition of the gut microbiota [19] as well as in the occurrence of the gastrointestinal tract and BSI [17, 18], including those caused by other MDRO such as methicillin-resistant *Staphylococcus aureus* (MRSA) [20].

Given the increasing relevance of VRE as emerging MRO worldwide [10], we sought to assess the existence of sex differences in VRE BSI at our institution by analyzing patient data collected between 2015 and 2020. This showed a male bias in the occurrence of VRE bacteremia, with male patients accounting for 73% (*n*=45) of all cases of VRE BSI (*n*=71) in this period. Thereupon, we conducted a systematic review of evidence available in digital databases, focusing on bacteremia as indicator infection with the aim of facilitating comparability with current epidemiological data worldwide.

## Main text

This systematic review with meta-analysis was planned and conducted according to the American Medical Association standards for meta-analysis of observational studies in epidemiology (MOOSE) [21] and the Preferred Reporting Items for Systematic Reviews and Meta-Analyses (PRISMA) guidelines [22].

### Search strategy and inclusion/exclusion criteria

Queries of literature were performed with the help of the electronic databases PubMed and Web of Science until May 2020 using the following combination of search terms to identify relevant articles: ((‘vancomycin resistant enterococci’) OR (‘vancomycin resistant enterococcus’) OR VRE) AND ((‘blood stream infection’) OR (‘bacteremia’) OR (‘bacteraemia’)) AND (male OR female OR sex OR gender). The literature search was conducted by two authors (CC and SK), with discrepancies resolved via discussion. Search results were limited to English language articles with a start date on 01/01/2015. Articles were included if they were conducted in hospitalized patients suffering from VRE BSI with an assumingly balanced sex ratio. Articles were excluded if they did not contain original peer-reviewed research (e.g., case reports, review articles, letters, etc.) and if no balanced sex ratio in patients could be assumed (e.g., veteran hospital studies, obstetric patients’ cohort).

### Data extraction and risk of bias assessment

After determination of the study selection, two investigators independently extracted the following characteristics: the first author’s last name, year of publication, study origin, patient clientele, sample size, VRE BSI sample size, and prevalence of VRE BSI among observed male and female patients. To assess the risk of bias and quality of the included studies, we used the Cochrane risk of bias tool for prognosis studies QUIPS, which grades studies on a scale from high risk to low risk of bias due to study participation, study attrition, prognostic factor measurement, outcome measurement, study confounding, and statistical analysis and reporting. For each factor, 1 score point was assigned, resulting in a maximum of 6 points if no bias was detected. For partial bias, 0.5 points and for reliable bias 0 points were assigned, respectively.

### Statistical analysis

Proportions and 95% CI were calculated for each study, with VRE BSI being the outcome variable and male sex representing the exposure. The pooled proportion was estimated using a random effects model with DerSimonian and Laird as variance estimator. Statistical inconsistency test *I*^2^ was used to assess inter-study heterogeneity, which was assumed at *I*^2^ > 50%. All the analyses were made using the software R version 3.6.3 (R Foundation for Statistical Computing, Vienna, Austria). Visualization of data was performed using the forest-plot package of the same software.

### Results

**Fig. 2:**
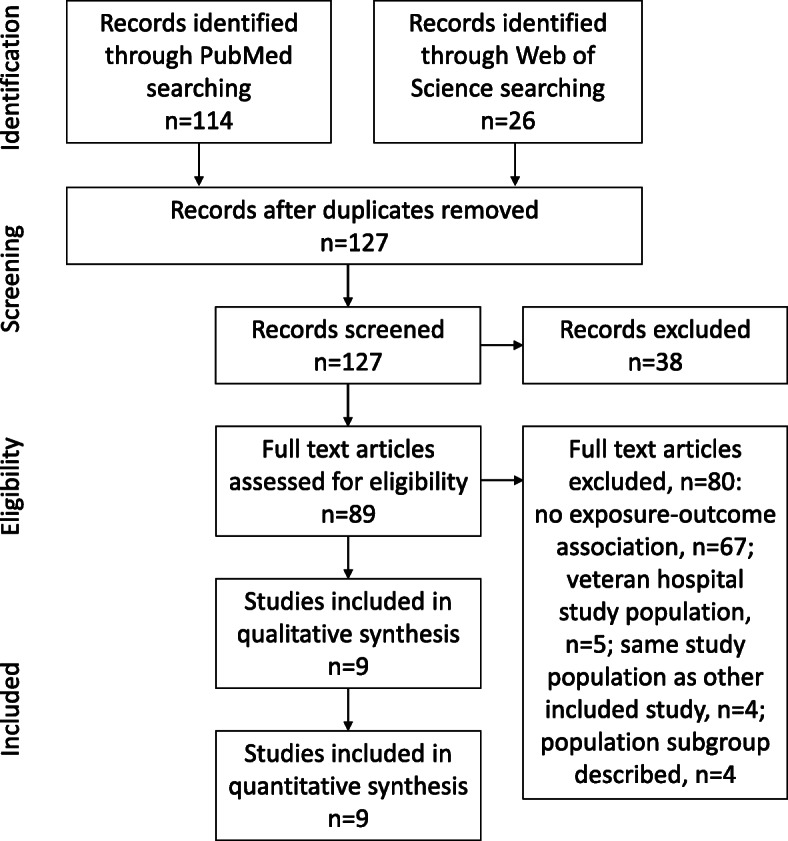
Systematic review flow chart

The literature screening process revealed nine articles [23–31], which were included for quantitative synthesis of data and are discussed in this review. Figure 2 summarizes the full screening and inclusion procedure. Included articles comprise cohort [23], cross-sectional [23], chart review [25], and case-control studies [27, 29], mostly with retrospective nature of conducted analyses from eight different countries. All admitted patients were considered in six of nine studies. In three studies [24, 26, 31], the analyzed population was limited due to age and/or underlying diagnoses. Further characteristics from each study can be extracted from Table 1.

**Table 1:** Characteristics of included studies

Study	Design	Location	Duration	Patient population	Sample size (male sex %)	Patients with VRE BSI	VRE BSI sex ratio (m:f)
Chen et al. [23]	Retrospective cross-sectional study	Changhua, Taiwan	2010–2014	All admitted patients with enterococcal BSI	279 (NM)	36	4:5
Ford et al. [24]	Cohort study	Salt Lake City, USA	2006–2014	Admitted HSCT patients with enterococcal BSI	161 (72%)	10	1:1
Xie et al. [25]	Retrospective chart review	Melbourne, Australia	2008–2013	All admitted patients with VRE BSI	96 (52%)	96	10:9
Ye et al. [26]	Retrospective cohort study	Taoyuan, Taiwan	2011–2015	Admitted patients > 18 years with VRE BSI	210 (53%)	210	10:9
Johnstone et al. [27]	Matched case-control study	Ontario, Canada	2009–2013	All admitted patients with VRE BSI matched to 3 controls	868 (60%)	217	3:2
Ryan et al. [28]	Retrospective cohort study	Dublin, Ireland	2009–2012	All admitted patients with VRE BSI	75 (60%)	75	3:2
Gouliouris et al. [29]	Nested case-control study	Cambridge, UK	2006–2012	Admitted patients with VRE BSI matched 1:1 to controls	455 (60%)	235	8:5
Kramer et al. [30]	Retrospective cohort study	Berlin, Germany	2008–2015	All admitted patients with enterococcal BSI	1160 (61%)	103	2:1
Bae et al. [31]	Retrospective cohort study	Seoul, Korea	2010–2017	Admitted patients ≤ 18 years with underlying malignancies and enterococcal BSI	30 (70%)	11	9:2

*NM* not mentioned, *m* male, *f* female

Risk of bias assessment revealed five studies with 6 points [25, 27–30], indicating no risk of bias. The minimum score achieved was 4 points in two of the included studies, indicating a moderate risk of bias according to the chosen QUIPS tool. Details of the risk of bias assessment can be gathered from Table 2.

**Table 2:** Risk of bias assessment

Domain	Potential bias	Studies
1. Study participation	Yes Partly No Unsure	Ford et al., Bae et al. Ye et al. Chen et al., Xie et al., Johnstone et al., Ryan et al., Gouliouris et al., Kramer et al. None
2. Study attrition	Yes Partly No Unsure	None Chen et al. Ford et al., Xie et al., Ye et al., Johnstone et al., Ryan et al., Gouliouris et al., Kramer et al., Bae et al. None
3. Prognostic factor measurement	Yes Partly No Unsure	None None Chen et al., Ford et al., Xie et al., Ye et al., Johnstone et al., Ryan et al., Gouliouris et al., Kramer et al., Bae et al. None
4. Outcome measurement	Yes Partly No Unsure	None None Chen et al., Ford et al., Xie et al., Ye et al., Johnstone et al., Ryan et al., Gouliouris et al., Kramer et al., Bae et al. None
5. Confounding measurement and account	Yes Partly No Unsure	Ford et al., Bae et al. Ye et al. Chen et al., Xie et al., Johnstone et al., Ryan et al., Gouliouris et al., Kramer et al. None
6. Analysis	Yes Partly No Unsure	None None Chen et al., Ford et al., Xie et al., Ye et al., Johnstone et al., Ryan et al., Gouliouris et al., Kramer et al., Bae et al. None

**Fig. 3:**
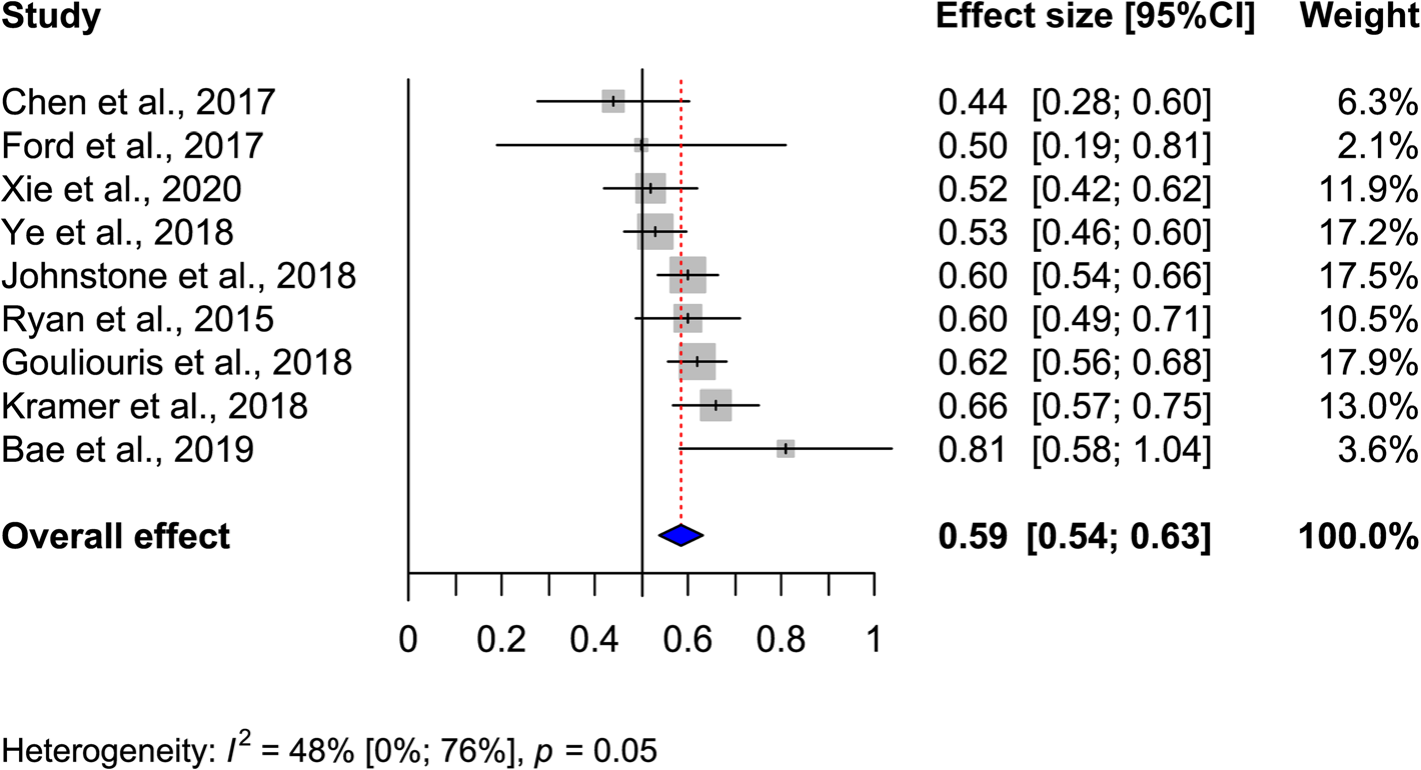
Forest plot displaying included articles

All the nine selected studies contributed to the statistical analysis. As there was a borderline heterogeneity in selected studies (*I*^2^ = 48% [0%;76%]; *p*=0.05), a random effects model was employed. In a single study [23], sex ratio favors female patients for VRE BSI. All other eight studies revealed a gender bias towards male sex (Table 1, Fig. 2). The pooled effect size revealed 59% patients suffering from VRE BSI to be of male sex resulting in a pooled prevalence male/female ratio of 1.4 (Fig. 3).

### Discussion

VRE are worldwide spread MDRO that thrive in healthcare settings and can cause severe invasive infections such as bacteremia. The evidence analyzed in this study indicates sex differences with male preference (59%) in the distribution of VRE BSI cases. This is in accordance with data collected during a 5-year period at our own institution, a university hospital with 1500 beds. Furthermore, epidemiological trends observed in the German federal state in which our hospital is located, a region with over 20 million inhabitants, show that 61% of all patients with VRE bacteremia between 2016 and 2019 were male (Correa-Martínez et al., unpublished data).

Sex differences have been previously reported in population-based studies on BSI caused by several pathogens, including *Staphylococcus aureus* (male bias) [20, 32] and *Escherichia coli* (female bias) [32, 33]. Besides socioeconomic, behavioral, and other contextual factors that could contribute to this phenomenon, certain biological determinants are considered to play a role in the sex differences observed in infectious diseases on the epidemiological level (Fig. 1). These include genetic and hormonal factors.

A particular characteristic of the XX genotype is the inactivation of parts of the X chromosomes. The resulting mosaicism leads to a transcriptional silencing of genes encoding chromosomal immune defects such as the X-linked chronic granulomatous disease and the X-linked Wiskott-Aldrich syndrome [34]. Moreover, the X chromosome also encodes microRNAs bearing an important immunoregulatory function [35].

The influence of sex steroid hormones on the immune response has been well documented [17, 34]. While higher levels of anti-inflammatory cytokines such as IL-10 have been detected in females with sepsis, pro-inflammatory cytokines IL-6 and TNF-α seem to predominate in males [36, 37]. Inflammation and tissue injury might also be enhanced by testosterone through stimulation of neutrophil activation [38]. However, estrogen has also been found to promote inflammatory responses, enhancing natural killer (NK) cell cytotoxicity and inducing the production of IL-1, Il-6, and TNFα [39, 40]. Furthermore, testosterone may induce immunosuppression by decreasing the expression of the major histocompatibility complex (MHC) class II and the toll-like receptor 4 (TLR4) on immune cells [40, 41]. Taken together, data indicate that sex hormones decisively influence immunity not by inducing a sex-specific pro- or anti-inflammatory effect, but rather by affecting the balance between both states in response to infectious agents. The immune homeostasis regulation seems to be more effective in females, as shown by the higher rate of splenocyte proliferation and production of IL-2 and IL-3 observed in animal models [42]. This leads to the belief that male immune response to sepsis can be more pronounced and prolonged, potentially inducing systemic damage more often than in females.

Female enhanced protection against microorganisms has been documented, as in the case of estrogen-driven, innate antibody-mediated immunological responses supporting clearance of enteropathogenic *Escherichia coli* from the bloodstream in animal models [43]. However, complex interactions between sex-specific and pathogen-specific immune responses may ultimately be decisive for outcomes in infection [44].

## Perspectives and significance

Although current evidence points towards a male bias in VRE bacteremia, data is scattered and systematic assessment of evidence regarding this phenomenon is lacking. Our review constitutes a first comprehensive approach to the evidence available on the sex distribution of VRE bacteremia, highlighting consistent sex differences among published studies with male patients developing this condition significantly more frequently than female patients do.

The evidence reviewed in this work does not allow to establish causality. It rather indicates a significant association between male sex and occurrence of VRE bacteremia, a condition that can partially be favored by confounders such as underlying pathologies or antibiotic administration that predispose for susceptibility. The establishment of causal relationships through the characterization of causal (e.g., immunomodulatory) pathways and other factors involved warrants further epidemiological and biomedical research.

Sex differences regarding the outcomes of VRE BSI and possible discrepancies with the observed male predominance in the development of VRE bacteremia constitute a further research topic, considering evidence indicating a higher female mortality in spite of a lower incidence of MRSA BSI [45, 46].

In light of the growing challenges posed by VRE to healthcare systems worldwide, evidence on sex differences of invasive VRE infections constitutes valuable information at the clinical, epidemiological, and policymaking levels.

## Data Availability

The datasets used and/or analyzed during the current study are available from the corresponding author on reasonable request.
